# Transcript levels of spindle and kinetochore-associated complex 1/3 as prognostic biomarkers correlated with immune infiltrates in hepatocellular carcinoma

**DOI:** 10.1038/s41598-021-89628-z

**Published:** 2021-05-27

**Authors:** De-Chen Yu, Xiang-Yi Chen, Xin Li, Hai-Yu Zhou, De-Quan Yu, Xiao-Lei Yu, Yi-Cun Hu, Rui-Hao Zhang, Xiao-Bo Zhang, Kun Zhang, Jiang-Dong An

**Affiliations:** 1 Department of Orthopedics, Lanzhou University Second Hospital, Lanzhou, 730000 China; 2 Department of Orthopedics, Xigu Branch of the Second Hospital of Lanzhou University, Lanzhou, 730000 China; 3 Department of Radiotherapy, Air Force Medical University Tangdu Hospital, Xi’an, 710000 China; 4 Department of Cardiology, Air Force Medical University Tangdu Hospital, Xi’an, 710000 China

**Keywords:** Cancer, Computational biology and bioinformatics, Immunology, Biomarkers, Oncology

## Abstract

The spindle and kinetochore-associated protein complex (Ska) is an essential component in chromosome segregation. It comprises three proteins (Ska1, Ska2, and Ska3) with theorized roles in chromosomal instability and tumor development, and its overexpression has been widely reported in a variety of tumors. However, the prognostic significance and immune infiltration of Ska proteins in hepatocellular carcinoma (HCC) are not completely understood. The bioinformatics tools Oncomine, UALCAN, gene expression profiling interactive analysis 2 (GEPIA2), cBioPortal, GeneMANIA, Metascape, and TIMER were used to analyze differential expression, prognostic value, genetic alteration, and immune cell infiltration of the Ska protein complex in HCC patients. We found that the mRNA expression of the Ska complex was markedly upregulated in HCC. High expression of the Ska complex is closely correlated with tumor stage, patient race, tumor grade, and TP53 mutation status. In addition, high expression of the Ska complex was significantly correlated with poor disease-free survival, while the high expression levels of Ska1 and Ska3 were associated with shorter overall survival. The biological functions of the Ska complex in HCC primarily involve the amplification of signals from kinetochores, the mitotic spindle, and (via a MAD2 invasive signal) unattached kinetochores. Furthermore, the expression of the complex was positively correlated with tumor-infiltrating cells. These results may provide new insights into the development of immunotherapeutic targets and prognostic biomarkers for HCC.

## Introduction

Hepatocellular carcinoma (HCC), the most common type of primary liver cancer, is one of the most common cancers and ranks second among causes of cancer-related deaths^1^. Hepatitis virus infection, alcohol consumption, obesity, and aflatoxin are considered risk factors for HCC^2,3^. In recent years, the treatment of liver cancer has been greatly developed, including arterial chemoembolization, hepatectomy, radiotherapy, and targeted therapy. However, due to frequent late-stage diagnosis, recurrence, and metastasis, the overall 5-year survival rate (7%) remains poor^4^. Many studies have explored the role of immune infiltration-related mechanisms in HCC, in search of specific targets for immunotherapy^5^. Thymocyte selection-associated high mobility group box protein (TOX) and P-selectin glycoprotein ligand-1 (PSGL-1) modulate the tumor microenvironment by depleting CD8 + T cells, and they hold promise as targets for tumor immunotherapy^6,7^. Due to the poor treatment results and low survival rate of HCC, it is particularly important to identify reliable predictive biological targets for early diagnosis and to improve the prognosis of patients through immunotherapy.

Mitotic abnormalities are a common feature of most tumors, and the separation of chromosomes during mitosis is mainly driven by kinetochores attached to specific regions of spindle microtubules^8^. The spindle and kinetochore-associated (Ska) complex is composed of three protein subunits: Ska1, Ska2, and Ska3, which are necessary for the stabilization of kinetochore-spindle microtubule attachment during mitosis^9,10^. Many studies have shown that dysregulation of the *SKA* family of genes is associated with a variety of cancers. For example, upregulation of *SKA1* expression in esophageal squamous cell carcinoma tissues is associated with tumor differentiation and pathological tumor node metastasis (TNM) stage. Esophageal cancer patients with high expression of *SKA1* have a poorer prognosis than patients with low expression^11^. *SKA2* is significantly upregulated in breast cancer tissues and is associated with TNM stage and lymph node metastasis. High expression of *SKA2* promotes invasion and metastasis of breast cancer cells via epithelial–mesenchymal transition (EMT)^12^. *SKA3* expression is also increased in cervical cancer tissues, and cervical cancer patients with high *SKA3* expression have a poor prognosis. *SKA3* overexpression promotes cervical cancer cell proliferation and migration and accelerates tumor growth^13^. In pancreatic cancer, high expression of *SKA1* and *SKA3* is associated with poor prognosis and immune cell infiltration^14^. Furthermore, SKA1 has emerged as a prognostic indicator associated with tumor cell infiltration and holds promise as a therapeutic target in adrenocortical carcinoma^15^.

In recent years, the SKA gene family has been increasingly studied in HCC, and previous studies have shown that these genes are highly expressed in HCC^16–18^. However, the potential importance of SKA genes in this disease, especially in prognostic development and immune infiltration, has not been comprehensively elucidated.

The role of the SKA gene family in HCC has been explored with gene sequencing and the use of various bioinformatics databases. In this study, several databases were used for data mining of HCC patients, aiming to systematically and comprehensively explore the gene expression, prognostic value, immune correlation, and potential function of SKA genes in HCC patients. Our study may reveal the molecular mechanisms involved in the expression and regulation of the Ska complex and the development of HCC and could provide reliable targets for HCC diagnosis and treatment.

## Materials and methods

### Oncomine database

The Oncomine database (www.oncomine.org) is a publicly accessible online database that provides an analysis of genome-wide expression with a range of cancer microarray information^19^. The expression data of SKA genes in diverse cancer types were obtained from Oncomine. In this study, a Student’s t-test was performed on this data with the significance threshold set as follows: *P* value = 0.05; fold change = 2; gene rank: 10%; data type: mRNA.

### UALCAN

UALCAN (http://ualcan.path.uab.edu/analysis.html) is an interactive web resource for in-depth analysis of cancer data from The Cancer Genome Atlas (TCGA) database^20^. It was used to analyze the expression of *SKA1-3* in both normal and cancerous tissues. Student’s t-test was used to generate *P* values. The *P* value cutoff was set at 0.05.

### GEPIA2

GEPIA2 (http://gepia2.cancer-pku.cn/) is a website for analyzing the RNA sequencing expression data of 9736 tumors and 8587 normal samples from the TCGA and Genotype-Tissue Expression (GTEx) projects, using a standard processing pipeline^21^. In this study, we explored the expression differences of SKA genes in HCC tissues and normal tissues, the analysis of pathological stages, and the related prognostic analysis using the “Single Gene Analysis” module of GEPIA. Student’s t-test was used with a critical value for the *P* value of 0.05.

### cBioPortal

cBioPortal (www.cbioportal.org), an open online tool, can visualize and analyze multidimensional cancer genomics^22,23^. Based on the TCGA database, genetic alterations and the summary of gene types were analyzed using cBioPortal, as well as the relationship between gene mutations and the prognosis of HCC patients. Statistical significance was set at *P* < 0.05.

### GeneMANIA

GeneMANIA (http://www.genemania.org) is a flexible web interface that can generate hypotheses about gene function, analyze gene lists, and prioritize genes for functional assays^24^. Using GeneMANIA, it was possible to identify the relationships between the Ska proteins and their interactive genes.

### Metascape

Metascape (http://metascape.org) is a web-based portal that provides gene annotation, enrichment analysis, and a protein–protein interaction (PPI) network based on over 40 independent knowledgebases^25^. To verify the enrichment of the Ska proteins and the *SKA* genes, the “Express Analysis” module of Metascape was used.

### TIMER

TIMER (http://timer.cistrome.org/) is a freely available web server for investigating the infiltration of diverse immune cells and their clinical impact^26^. The Ska proteins were submitted to the “Gene module” of TIMER and their correlation with immune cells (B cells, CD4 + T cells, CD8 + T cells, macrophages, neutrophils, and dendritic cells) was explored.

## Results

### Abnormal expression of *SKA* genes in HCC patients

**Figure 1:**
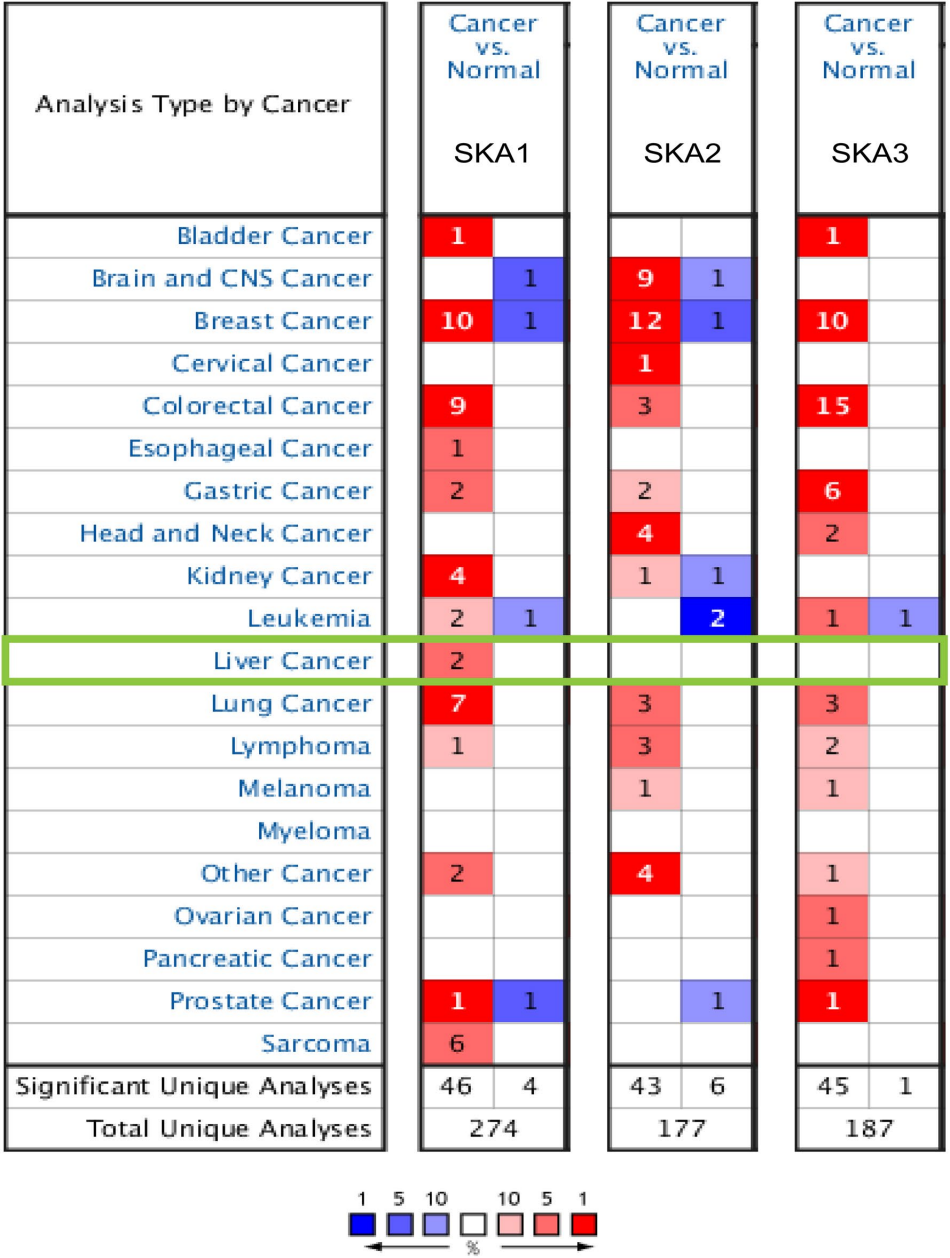
mRNA expression levels of SKAs in different types of human cancer (Oncomine). Red indicates high expression. Blue indicates low expression. *P* < 0.05, fold-change > 2 and gene rank = 10% were considered statistically significant. Numbers in each cell represent the data set meeting the threshold. *SKA* spindle and kinetochore-associated complex.

**Figure 2:**
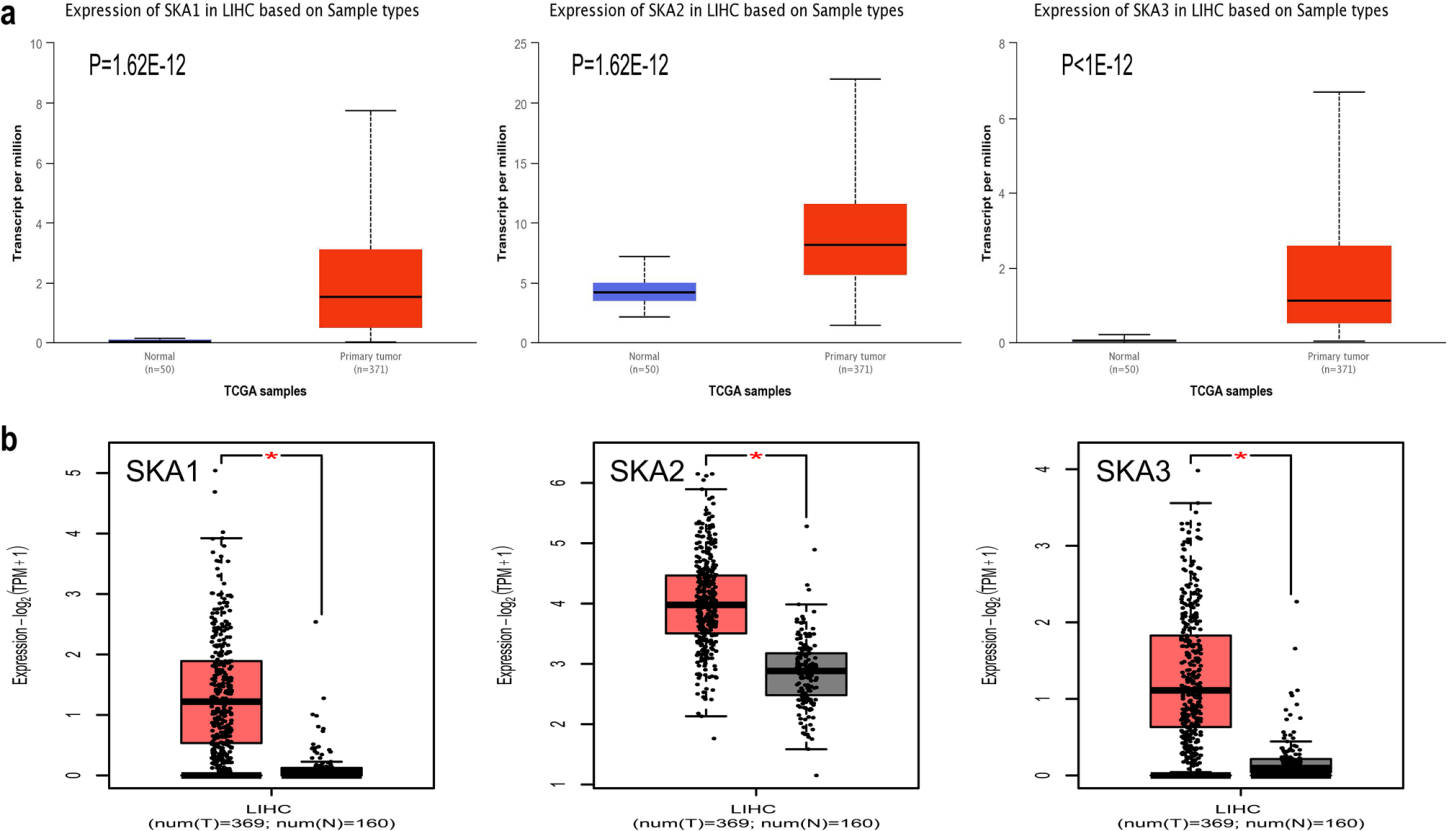
mRNA expression levels of SKAs in HCC patients. (**a**) mRNA expression levels of SKAs in HCC patients. (UALCAN). (red for tumor, blue for normal). (**b**) mRNA expression levels of SKAs in HCC patients. (GEPIA2). *P < 0.05. (red for tumor, black for normal). *HCC* hepatocellular carcinoma.

To explore the expression of *SKA* genes in HCC patients, we first quantified the mRNA expression level using the Oncomine database, which showed that *SKA1* expression was dramatically elevated in HCC tissues compared with normal tissues (Fig. 1). Specifically, using Chen’s dataset, the analysis showed that *SKA1* was overexpressed 2.534-fold in liver hepatocellular carcinoma (LIHC) specimens. Using the Wurmbach liver dataset (Table 1), *SKA1* was overexpressed 1.701-fold. UALCAN was also used to analyze the expression of *SKA* genes in HCC and normal tissues, and the results showed that the expression of *SKA1* (*P* = 1.62e−12), *SKA2* (*P* = 1.62e−12), and *SKA3* (*P* < 1e−12) increased significantly in HCC tissues (Fig. 2a). We also used the GEPIA2 database to verify the expression of *SKA* genes in HCC tissues. Consistent with the previous results, the protein expression levels of *SKA1*, *SKA2*, and *SKA3* were significantly higher in HCC patients (Fig. 2b). In summary, all three genes in the *SKA* family were significantly upregulated in HCC patients.

**Table 1:** Transcription expression of SKAs family members between LIHC and normal liver tissues (Oncomine).

Gene	Types of LIHC VS. liver	Fold change	*P* value	t-test	References
SKA1	Hepatocellular carcinoma	2.534	1.85E−12	7.518	Chen Liver^27^
	Hepatocellular carcinoma	1.701	1.98E−4	3.871	Wurmbach Liver^28^

### Clinicopathological parameters of *SKA* genes in HCC patients

**Figure 3:**
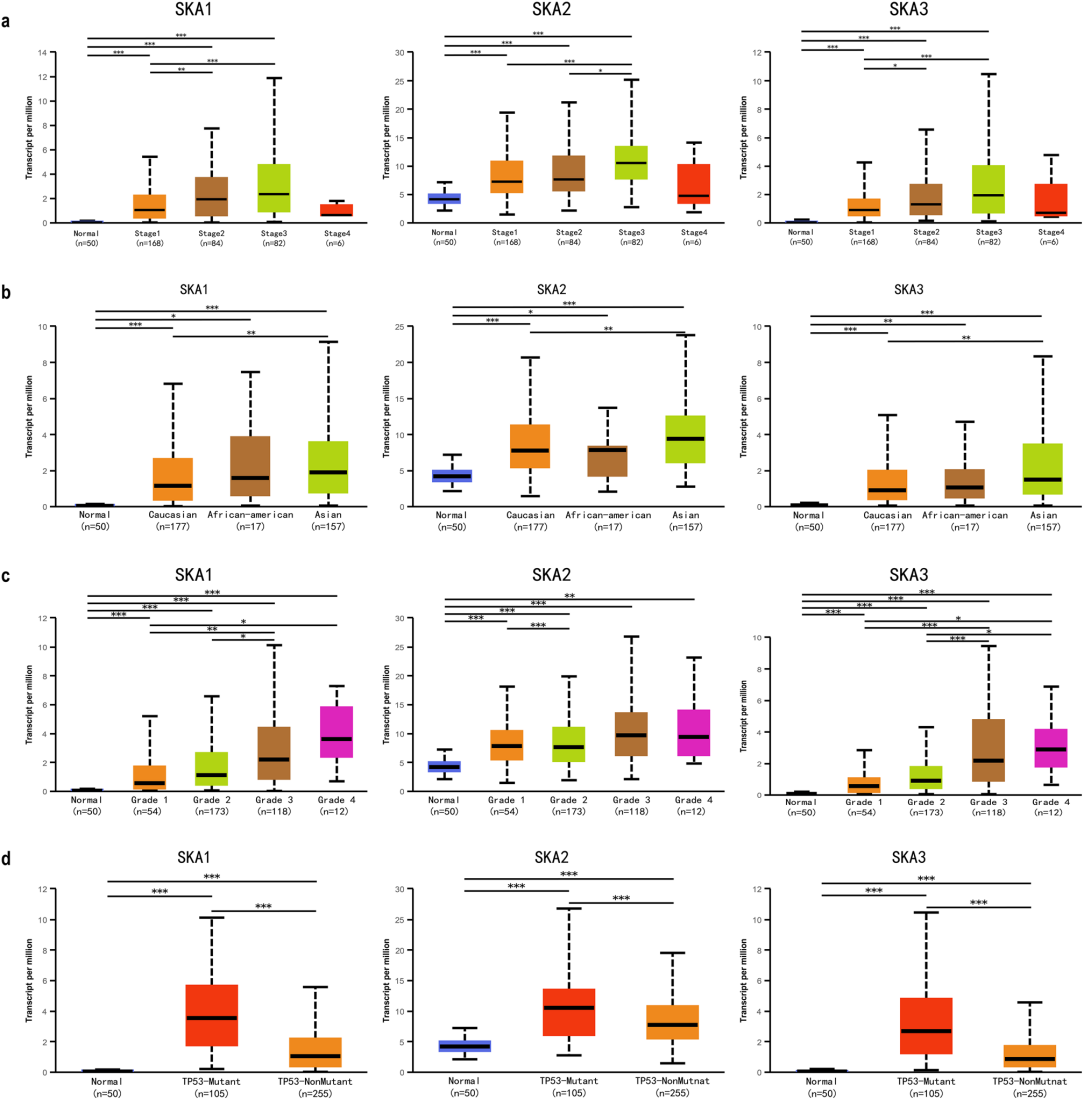
Correlation between mRNA expression levels of SKAs and clinicopathological parameters of HCC patients. (UALCAN). (**a**) Correlation between mRNA expression levels of SKAs and tumor stages of HCC patients. (**b**) Correlation between mRNA expression levels of SKAs and race of HCC patients. (**c**) Correlation between mRNA expression levels of SKAs and tumor grade of HCC patients. (**d**) Correlation between mRNA expression levels of SKAs and TP53 mutation status of HCC patients. *P < 0.05, **P < 0.01, **P < 0.001.

We explored the relationship between the *SKA* gene expression level and the clinical characteristics of patients with HCC, including tumor stage, patient race, tumor grade, and TP53 mutation status. As shown in Fig. 3a, *SKA* gene expression was significantly associated with the stage of HCC, with increased expression correlating with a higher stage. The expression of *SKA* family genes was significantly upregulated in stages 1, 2, 3, and 4 compared with normal liver tissue, while there was no significant difference between normal tissue and stage 4 HCC, possibly due to the small number of stage 4 cases (n = 6). Among these, there were also significant differences in the expression of *SKA* genes between stages 1 and 2 and between stages 2 and 3. We also examined the relationship between *SKA* expression levels and the race of the patients, which showed that Caucasian, African American, and Asian patients all presented with significantly higher expression levels compared with normal tissues, and that Asian patients showed higher expression differences than Caucasian patients (Fig. 3b). In terms of tumor grade, an increasing grade correlated with increased *SKA* gene expression (Fig. 3c). Furthermore, there was significant variability in the expression of *SKA* genes between levels. Finally, we also examined the relationship between the expression of *SKA* genes and the mutation status of TP53, which showed that expression of the Ska protein complex was significantly upregulated in patients with TP53 mutations compared with patients without mutations (Fig. 3d). In general, *SKA* gene expression levels were significantly correlated with tumor stage, tumor grade, and TP53 mutation status, and Asian patients presented a higher relative increase in the expression of *SKA* genes than Caucasian and African American patients.

### Prognostic value of *SKA* genes in HCC patients

**Figure 4:**
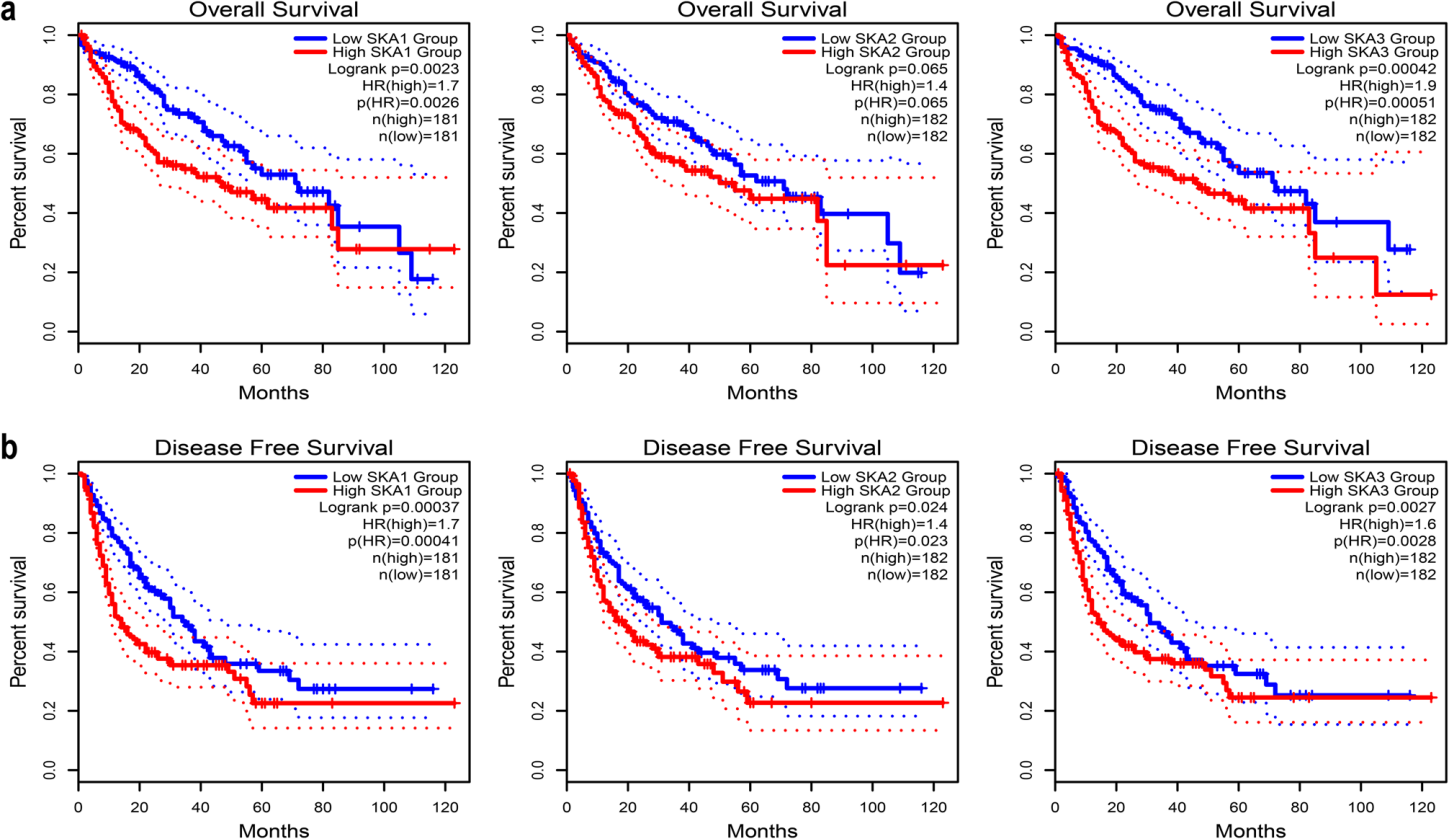
The prognostic value of mRNA expression level of SKAs in HCC patients (GEPIA2). (**a**) The relationship between SKAs expression and OS in HCC patients. (**b**) The relationship between SKAs expression and DFS in HCC patients. *OS* overall survival; *DFS* disease-free survival.

To determine the relationship between differential expression of *SKA* genes and prognosis of HCC patients, we first analyzed the correlation between differential expression and overall survival using GEPIA2. This showed that patients with high expression of *SKA1* (*P* = 0.0023) and *SKA2* (*P* = 0.00042) were primarily associated with shorter overall survival (Fig. 4a). The relationship between the differential expression of *SKA* genes and disease-free survival was also evaluated. We found that patients with high expression of *SKA1* (*P* = 0.00037), *SKA2* (*P* = 0.024), and *SKA3* (*P* = 0.0027) were significantly associated with shorter disease-free survival (Fig. 4b). These results suggest that *SKA* gene expression plays a crucial role in the prognosis of patients with HCC and may become a reliable predictor of survival in these patients.

### Frequency changes of *SKA* genes in HCC patients

**Figure 5:**
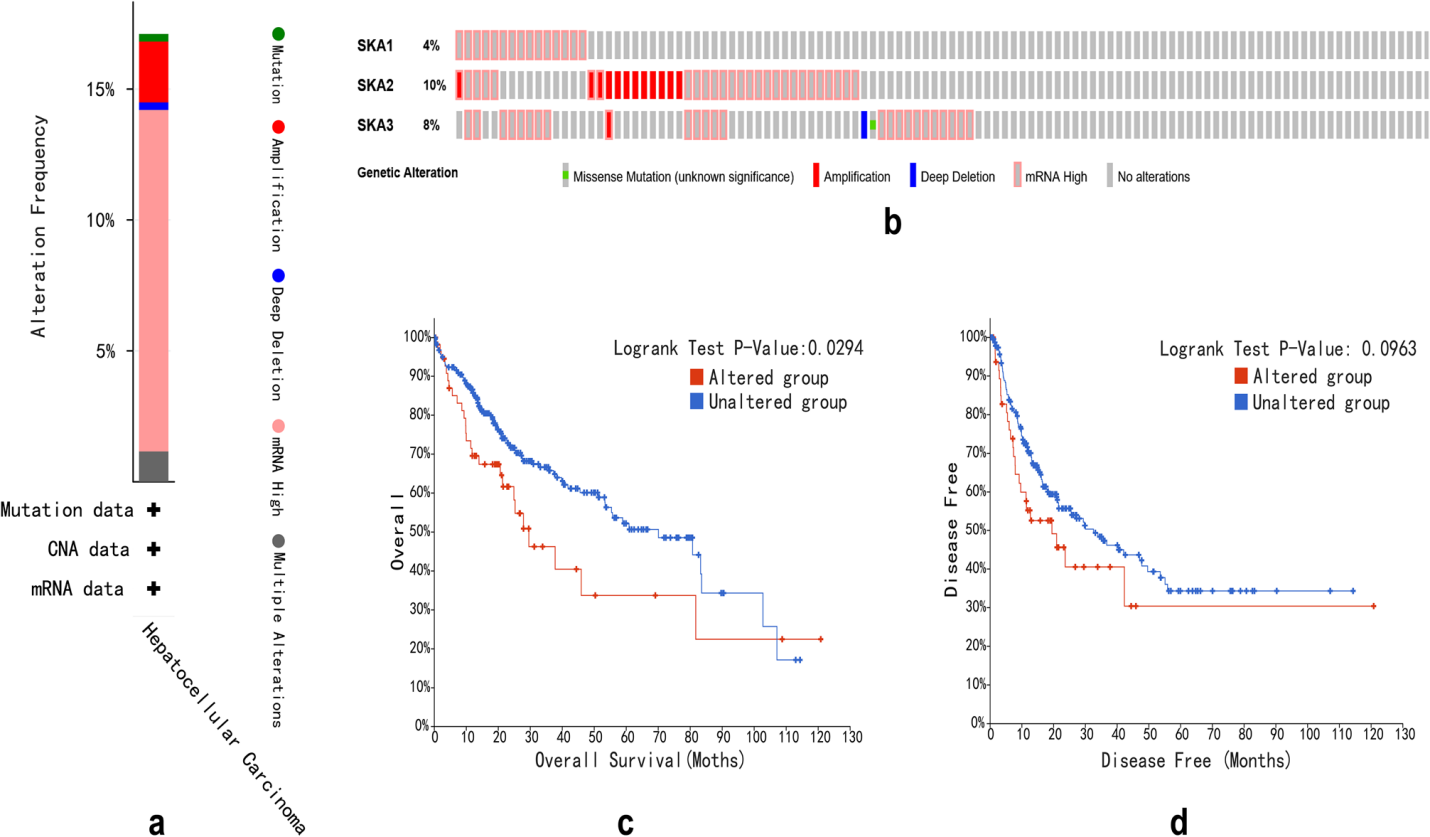
Alteration frequency of SKAs and their prognostic value in HCC patients (cBioPortal). (**a**,**b**) Summary of alterations in the SKAs in HCC patients. (**c**,**d**) K–M plots curve of OS and DFS in HCC patients with/without the SKAs alterations.

We used the cBioPortal database to analyze frequency changes in *SKA* genes in HCC patients. Fifty-nine (17%) patients had significant alterations in the *SKA* genes, including missense mutations, amplifications, deep deletions, and transcriptional upregulation. Specifically, the percentages of gene alterations in *SKA1*, *SKA2*, and *SKA3* were 4%, 10%, and 8%, respectively (Fig. 5a,b). We further explored the impact of gene alterations in the *SKA* family on the prognosis of patients with HCC, which showed that patients with genetically altered HCC had shorter overall survival than patients with unchanged *SKA* genes (*P* = 0.0294). However, there was no relationship between the *SKA* family gene alterations and disease-free survival (*P* = 0.0963) in HCC patients (Fig. 5c,d).

### Co-expression and enrichment of *SKA* genes in HCC patients

**Figure 6:**
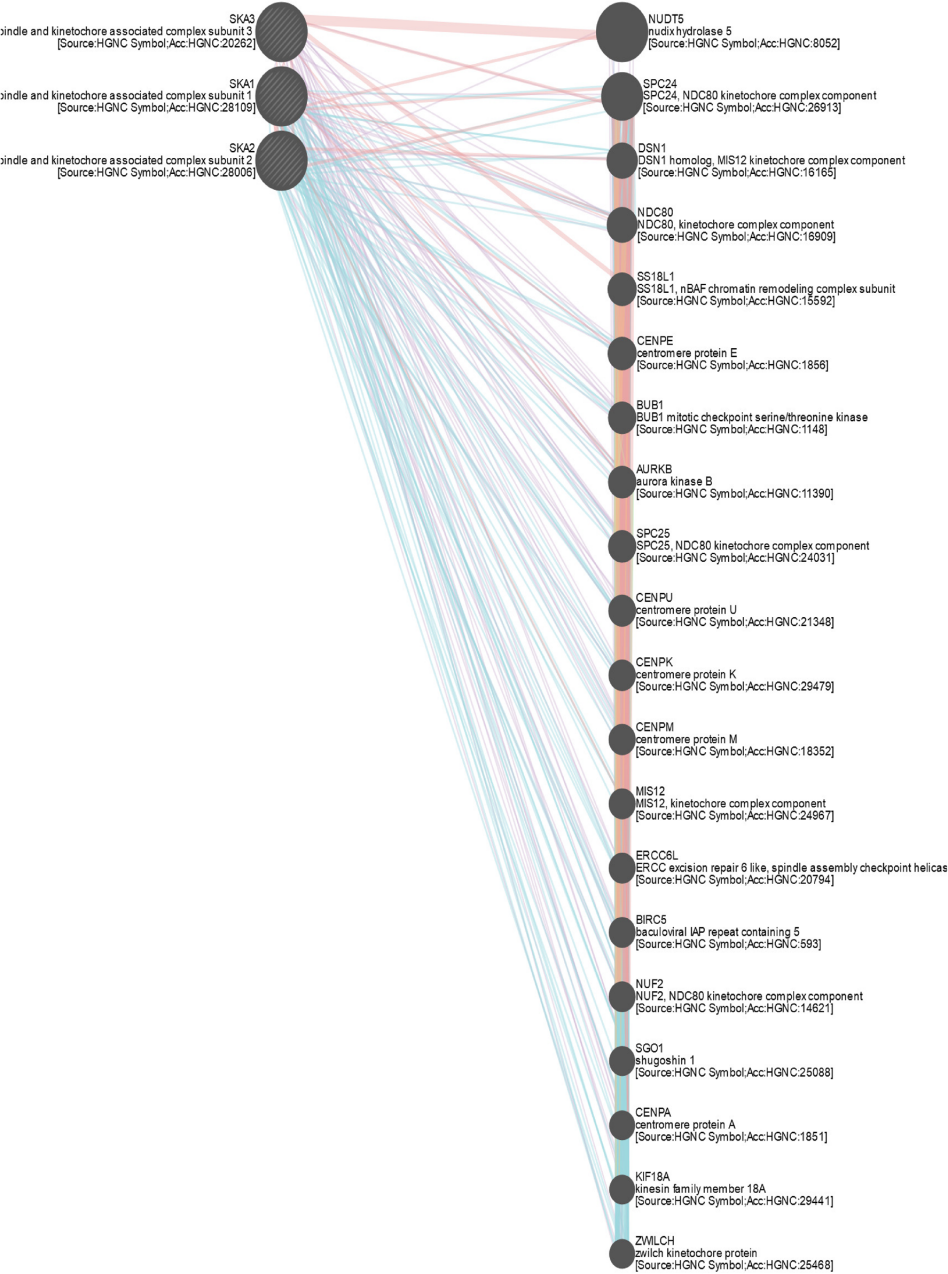
Gene–gene network of SKAs in HCC patients (GeneMANIA). GeneMANIA database identified 20 genes most associated with SKAs.

**Figure 7:**
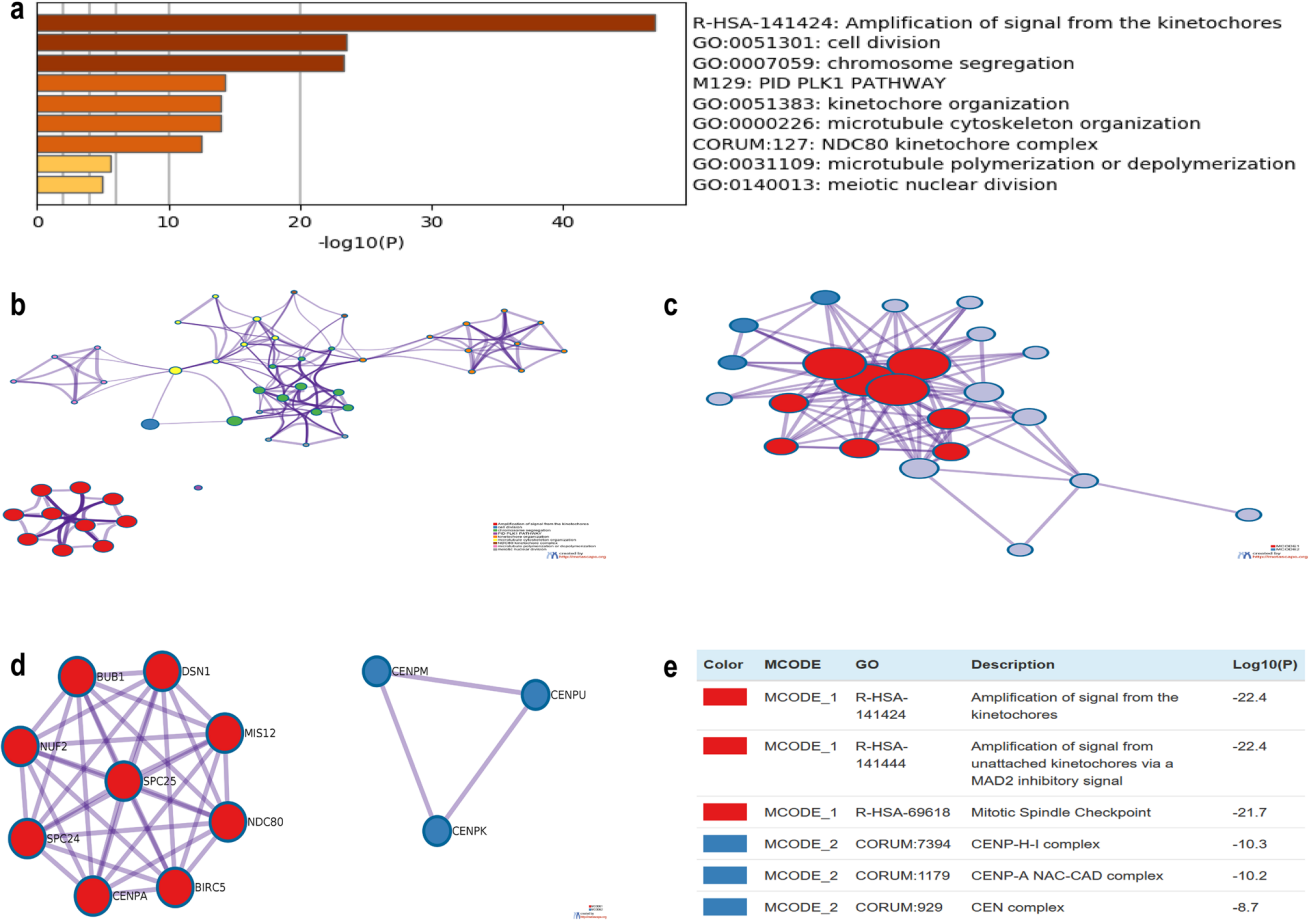
The enrichment analysis of SKAs and the 20 co-expressed genes in HCC patients (Metascape). (**a**) Bar chart of the first nine enriched terms for SKAs and the 20 co-expressed genes. (**b**) Net graph of enriched terms, Different colors represent different cluster ID. (**c**,**e**) PPI network and MCODE components identified.

To further explore the role of the *SKA* genes in HCC patients, we analyzed their regulatory network and their functionally similar genes using the GeneMANIA database. The results showed 20 genes with the strongest correlation, which were nudixhydrolase 5 (*NUDT5*), kinetochore protein SPC24 (*SPC24*), kinetochore-associated protein DSN1 homolog (*DSN1*), kinetochore protein NDC80 homolog (*NDC80*), SS18-like protein 1 (*SS18L1*), centromere protein E (*CENPE*), mitotic checkpoint serine/threonine-protein kinase BUB1 (*BUB1*), aurora kinase B (*AURKB*), kinetochore protein SPC25 (*SPC25*), centromere protein U (*CENPU*), centromere protein K (*CENPK*), centromere protein M (*CENPM*), protein MIS12 homolog (*MIS12*), DNA excision repair protein ERCC-6-like (*ERCC6L*), baculoviral IAP repeat containing protein 5 (*BIRC5*), kinetochore protein Nuf2 (*NUF2*), shugoshin 1 (*SGO1*), centromere protein A (*CENPA*), kinesin family member 18A (*KIF18A*), ZWILCH kinetochore protein (*ZWILCH*) (Fig. 6). Subsequently, we used Metascape to explore the biological functions of *SKA* genes and the aforementioned co-expressed genes. The top nine most abundant terms are shown in Fig. 7a: amplification of signals from the kinetochores, cell division, chromosome segregation, PID PLK1 pathway, kinetochore organization, microtubule cytoskeleton organization, NDC80 kinetochore complex, microtubule polymerization or depolymerization, and meiotic nuclear division. In addition, we constructed a network map of the enriched terms (Fig. 7b). To further analyze the relationship between *SKA* genes and HCC, PPI network maps were constructed, and MCODE component analysis was also performed (Fig. 7c–e). The most significantly different MCODE components were extracted from the PPI network graph, and the results showed that there were two significantly different components. MCODE1 was associated with amplification of signals from unattached kinetochores via a MAD2 inhibitory signal, amplification of signals from the kinetochores, and the mitotic spindle checkpoint. MCODE2 was associated with the CENP-H-I complex, CENP-A NAC-CAD complex, and CEN complex.

### Immune cell infiltration of Ska proteins in HCC patients

**Figure 8:**
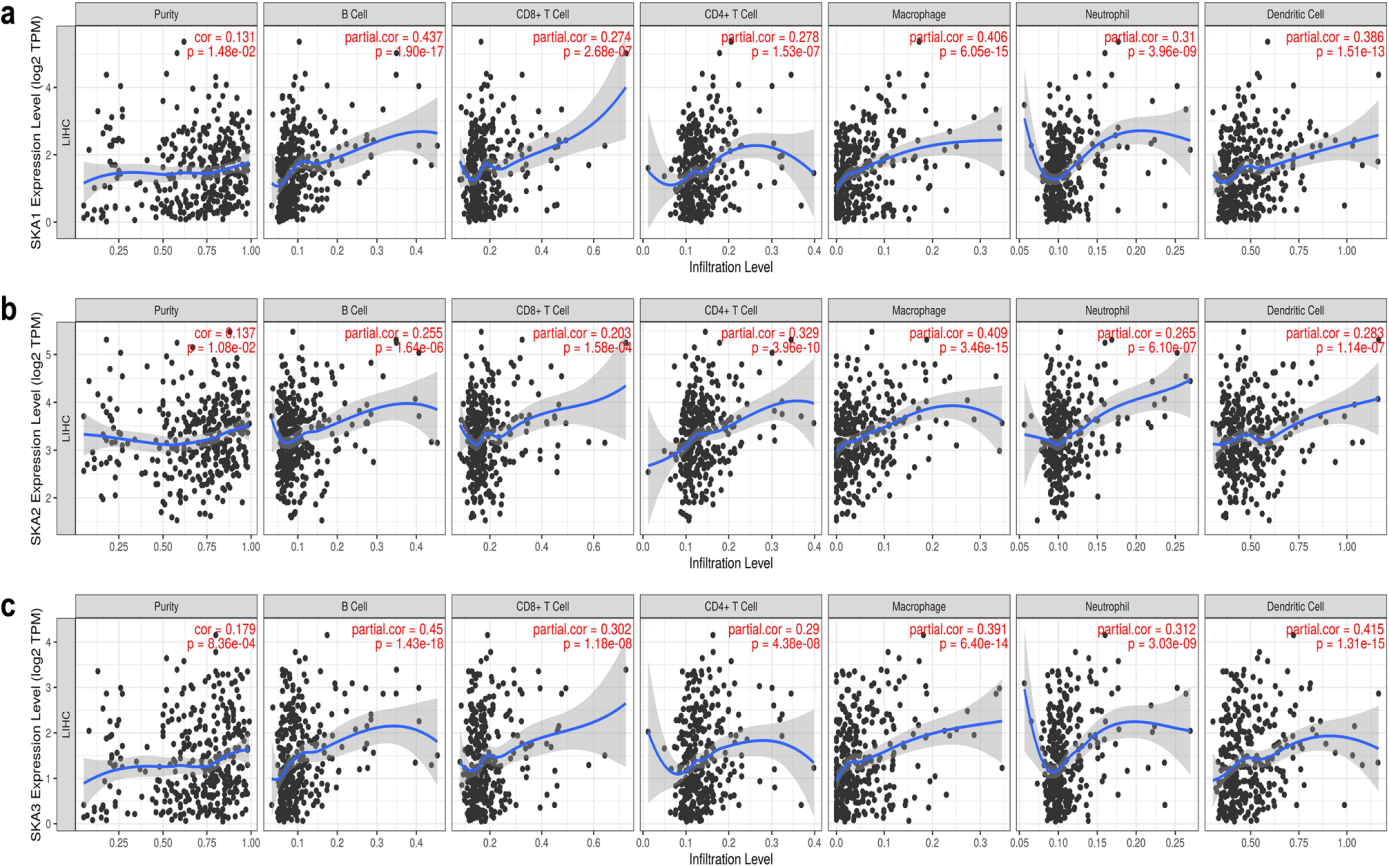
The relationship between SKAs and tumor immunological features of HCC patients. (**a**) The relationship between SKA1 and immune infiltrating cells. (**b**) The relationship between SKA2 and immune infiltrating cells. (**c**) The relationship between SKA3 and immune infiltrating cells.

Immune infiltration is associated with the development of cancer. In the present study, the TIMER database was used to analyze the correlation between *SKA* gene expression (and therefore Ska protein expression) and immune infiltration. All three *SKA* genes showed positive correlation between expression and immune cell infiltration. The expression of *SKA1* was positively correlated with B cells (Rho = 0.38, *P* = 2.90e−13), CD4 + T cells (Rho = 0.206, *P* = 1.16e−4), macrophages (Rho = 0.304, *P* = 8.41e−9), neutrophils (Rho = 0.412, *P* = 8.32e−3), and dendritic cells (Rho = 0.477, *P* = 4.93e−21) (Fig. 8a). *SKA2* was also positively correlated with B cells (Rho = 0.241, *P* = 5.97e−6), CD8 + T cells (Rho = 0.135, *P* = 1.23e−2), CD4 + T cells (Rho = 0.205, *P* = 1.3e−4), macrophages (Rho = 0.31, *P* = 34.29e−9), neutrophils (Rho = 0.237, *P* = 8.47e−6), and dendritic cells (Rho = 0.336, *P* = 1.47e−10) (Fig. 8b). Similarly, there was a positive correlation between the expression of *SKA3* and the infiltration of B cells (Rho = 0.42, *P* = 3.66e−12), CD4 + T cells (Rho = 0.237, *P* = 8.34e−6), macrophages (Rho = 0.298, *P* = 1.67e−8), neutrophils (Rho = 0.146, *P* = 6.43e−3), and dendritic cells (Rho = 0.498, *P* = 4.73e−23) (Fig. 8c).

## Discussion

Ska1, Ska2 and Ska3, which constitute the major components of the Ska complex, play a key role in the normal segregation of chromosomes during mitosis. Chromosomal malformation, a peculiar phenomenon of tumors, leads to genomic instability, thereby promoting tumor occurrence and development^29^. An increasing number of studies have demonstrated that Ska proteins play an important role in tumorigenesis, cancer cell proliferation, and apoptosis^30,31^. In recent years, immunotherapy has drawn increasing attention in the treatment of cancer; however, the prognostic value and immune infiltration of the Ska proteins in HCC have not been comprehensively explored.

This study demonstrated that the Ska proteins and the *SKA* genes were abnormally highly expressed in HCC, suggesting a link between the dysregulation of these genes and HCC. We further examined the relationship between the expression of *SKA* genes and the clinical characteristics of patients with HCC. The results showed that a higher expression of *SKA* genes was significantly correlated with tumor stage and pathological grade and that this expression also differed with the ethnicity of the patients, with a greater increase in expression levels in Asian than in Caucasian patients. In addition, there was a correlation between Ska protein complex expression and TP53 mutations. TP53 mutations are the most common mutations in HCC, and they lead to the downregulation of the immune response and differential expression of immune-related genes in HCC^32^. It has been shown that receptor activity-modifying protein 3 (RAMP3) in HCC patients may reduce the detrimental effect of TP53 mutations on survival^33^. Furthermore, in HCC patients, high expression of *SKA1* and *SKA2* was notably associated with shorter overall survival, while high expression of the Ska protein complex was markedly associated with shorter disease-free survival. To date, many studies have confirmed that *SKA* genes play an important role in HCC. One study examined 166 HCC and paired adjacent normal tissues and found that *SKA1* was highly expressed in HCC and correlated with tumor size and TNM stage^16^. Another study found that LINC00339 could interact with miR-1182 to promote the expression of *SKA1*, thereby accelerating the progression of HCC^34^. It has also been confirmed that *SKA2* can accelerate HCC progression by upregulating Wnt/β-catenin signaling^35^. Our findings regarding SKA gene expression in HCC are in agreement with those of a previous study. Therefore, we speculated that individual SKA genes or SKA family genes could serve as potential prognostic biomarkers for patients with HCC. However, the effects of SKA genes on the development, metastasis, cell proliferation, and apoptosis of HCC have not been comprehensively studied.

The occurrence and development of HCC is complex and multifaceted, and genetic changes play a role in this process^36^. Therefore, we explored the molecular characteristics of *SKA* genes in HCC. In HCC, the differential expression of *SKA* genes often undergo genetic changes, which are relevant to the overall survival rate. The most significant genetic change was the increase in mRNA expression.

Previous studies have revealed that Ska proteins function in other diseases, mainly by regulating chromosome segregation^37,38^. In this study, we explored the core genes underlying the function of Ska proteins, some of which have been identified as regulators of these proteins. Redli et al.^39^ reported that Ska proteins promote AURKB activity to limit their own microtubule and mitochondria association and ensure that kinetochore-microtubule (KT-MT) dynamics and stability fall within an optimal bi-directional equilibrium range. Sivakuma et al.^40^ demonstrated that NUF2 binding to Ska1 promotes the recruitment of the Ska complex to kinetochores, reducing metaphase arrest upon chromosome segregation. This implicates NUF2 as a potential regulator of Ska1. NDC80 has also been shown to influence the recruitment of the Ska complex^41^. In our study, we performed functional enrichment analysis to understand the biological functions of the *SKA* genes. The results suggest that these genes are mainly involved in the amplification of signals from the kinetochores and mitochondrial spindle checkpoint, and further amplification of signals from unattached kinetochores occurs via a MAD2 inhibitory signal. Our results concur with those of a previous report^42^ that high expression of the mitotic checkpoint protein MAD2 in the mammary gland of mice resulted in mitotic checkpoint hyperactivation, mitotic arrest, and retarded tumor growth. This suggests that *SKA* genes play a crucial role in the development and progression of tumors.

Accumulating evidence suggests that immune cell infiltration can influence tumorigenesis and recurrence and serve as an important determinant of immunotherapy response and clinical outcome^43^. Similarly, the immune microenvironment of tumors plays an important role in HCC^44^. CD8 + cytotoxic T lymphocytes can specifically identify major histocompatibility complex (MHC) antigens, which are widely used in tumor-targeted therapies^45^. One study showed that an elevated ratio of CD4+/CD8+ T cells was associated with a favorable prognosis in HCC^46^. It has also been shown that the co-expression of PD-1 and T-cell immunoglobulin and tyrosine inhibitory motif domain (TIGIT) in CD4+ and CD8+ T cells of HCC patients was significantly increased and negatively correlated with the overall survival and disease-free survival of patients^47^. Our study suggests that the expression levels of *SKA* genes may be significantly associated with the infiltration of immune cells (B cells, CD4+ T cells, macrophages, neutrophils, and dendritic cells). This suggests that *SKA* genes not only respond to the prognosis of HCC but also reflect the immune status of the disease and can provide new insights into HCC immunotherapy.

In addition, it must be acknowledged that our study has some limitations. First, we obtained data from several different databases, and it was difficult to guarantee that the data were consistent. Moreover, experimental validation of these data has not been performed at the time of writing, but this will be performed in our future work.

## Conclusions

We found that *SKA* gene expression levels were highly elevated in HCC. Ska1 and Ska3 could be considered potential prognostic markers. *SKA* gene expression was also significantly associated with the infiltration of immune cells (B cells, CD4 + T cells, macrophages, neutrophils, and dendritic cells), which indicated that Ska proteins may regulate the development of HCC by influencing the immune microenvironment. Inhibition of Ska protein expression and function, potentially in combination with immunotherapies, could represent a promising treatment strategy for patients with HCC.
